# Comparison of computed tomography and dual-energy X-ray absorptiometry in the evaluation of body composition in patients with obesity

**DOI:** 10.3389/fendo.2023.1161116

**Published:** 2023-06-26

**Authors:** Fiorella Palmas, Andreea Ciudin, Raul Guerra, Daniel Eiroa, Carina Espinet, Nuria Roson, Rosa Burgos, Rafael Simó

**Affiliations:** ^1^ Endocrinology and Nutrition Department, Hospital Universitari Vall D´Hebron, Barcelona, Spain; ^2^ Diabetes and Metabolism Research Unit, Vall d’Hebron Institut De Recerca (VHIR), Barcelona, Spain; ^3^ Department of Medicine, Universitat Autònoma De Barcelona, Barcelona, Spain; ^4^ Centro De Investigación Biomédica En Red De Diabetes y Enfermedades Metabólicas Asociadas (CIBERDEM), Instituto De Salud Carlos III (ISCIII), Madrid, Spain; ^5^ ARTIS Development, Las Palmas, Spain; ^6^ Department of Radiology, Institut De Diagnòstic Per La Imatge (IDI), Hospital Universitari Vall d’Hebron, Barcelona, Spain; ^7^ Nuclear Medicine Deparment, Vall Hebron Hospital, Barcelona, Spain

**Keywords:** obesity, morbid obesity, body composition, computed tomography, dual-energy X-ray absorptiometry

## Abstract

**Objective:**

a) To evaluate the accuracy of the pre-existing equations (based on cm2 provided by CT images), to estimate in kilograms (Kg) the body composition (BC) in patients with obesity (PwO), by comparison with Dual-energy X-ray absorptiometry (DXA). b) To evaluate the accuracy of a new approach (based on both cm2 and Hounsfield Unit parameters provided by CT images), using an automatic software and artificial intelligence to estimate the BC in PwO, by comparison with DXA.

**Methods:**

Single-centre cross-sectional study including consecutive PwO, matched by gender with subjects with normal BMI. All the subjects underwent BC assessment by Dual-energy X-ray absorptiometry (DXA) and skeletal-CT at L3 vertebrae. CT images were processed using FocusedON-BC software. Three different models were tested. Model 1 and 2, based on the already existing equations, estimate the BC in Kg based on the tissue area (cm2) in the CT images. Model 3, developed in this study, includes as additional variables, the tissue percentage and its average Hounsfield unit.

**Results:**

70 subjects (46 PwO and 24 with normal BMI) were recruited. Significant correlations for BC were obtained between the three models and DXA. Model 3 showed the strongest correlation with DXA (r= 0.926, CI95% [0.835-0.968], p<0.001) as well as the best agreement based on Bland – Altman plots.

**Conclusion:**

This is the first study showing that the BC assessment based on skeletal CT images analyzed by automatic software coupled with artificial intelligence, is accurate in PwO, by comparison with DXA. Furthermore, we propose a new equation that estimates both the tissue quantity and quality, that showed higher accuracy compared with those currently used, both in PwO and subjects with normal BMI.

## Introduction

Obesity is a chronic and relapsing disease which prevalence is significantly increasing worldwide and its related comorbidities suppose a high cost for healthcare system (1–3).

At present body mass index (BMI) remains a categorical diagnostic criterion for obesity. Nevertheless, BMI has serious limitations and do not provide information on the body composition (BC) and the metabolic condition of the subjects (4). Recently, efforts have focused to identify more specific prognostic factors and biomarkers of obesity and its metabolic complications (5–7). The American Association of Clinical Endocrinologists (AACE) proposed a new definition for obesity: “adiposity-based chronic disease (ABCD)”, which has also adopted by the European Society of Obesity (EASO) (8, 9). The concept “adiposity-based” refers not only to the total quantity of body fat, but also to its distribution and/or functionality (4, 10).

Furthermore, besides body fat, the muscle mass has a very relevant and complex role in the BC and body homeostasis and metabolic condition (11–13). Sarcopenia, which is the loss of muscle mass and strength of physical function synergistically worsen the adverse effects of obesity (14). However, the study of BC is crucial for identifying sarcopenia, especially in PwO, where body volume can mask low muscle mass if only anthropometric data is used for the clinical assessment (15). Recently, the European Society for Clinical Nutrition and Metabolism (ESPEN) and EASO have agreed a definition and diagnostic criteria for sarcopenic obesity, a condition in which sarcopenia and obesity coexists and leads to a cumulative risk derived from the two clinical situations (15, 16). In addition to measure the amount of adipose tissue or muscle mass, it is important to know its distribution and proportion, given its significant role in pathologies such as metabolic syndrome and associated complications (17–20). However, methods to assess BC are not taken into consideration in the daily clinical practice in the obesity management due to the lack of simple and reliable tests.

Dual-energy X-ray absorptiometry (DXA) has long been considered a reference technique to assess BC and it is still a reference method (14). It provides the mass of the different tissues, measured in kilograms. However, DXA does not provide information on the distribution of adipose tissue at the abdominal level (SAT or VAT) (21). Furthermore, in many clinics, accessibility to DXA is limited and except for the assessment of bone density, it is not a test that is performed in the usual clinical routine (22, 23).

In this scenario, CT emerges as a technique widely used in clinical practice that contains very precise information for assessing BC (24–28). Regional analysis of fat and fat -free mass at the third lumbar vertebra was shown to have a high correlation with total BC, and provides significant additional information of tissue quality and myosteatosis, based on the Hounsfield units (HU) (29–32). To obtain the CT image, the emission of radiation is necessary (approximately 10 mSv). Nonetheless, at present the assessment of BC by means of a CT image is been largely used in clinical research, especially in those pathologies in which the CT evaluation is part of the protocol, such as some types of cancer or abdominal pathologies (33).

Currently, BC assessment by CT is obtained by manual or semi-automatic marking software that provide an area parameter (cm^2^) and average of Hounsfield Units of the of target tissue (34). The use of this type of software requires trained staff able to manually correct the images, therefore, the evaluation of these results is time consuming and unfeasible in the current clinical practice at large scale. However, emerging technologies, such as artificial intelligence (AI), have promoted the development of new software tools able to rapidly and precisely analyze the images obtained by CT resulting in qualitative and quantitative information (24, 25, 27, 35). As far as we know at present no such tool coupled with AI is used for the BC assessment based on CT scan images in PwO.

The accuracy of the BC assessment by CT image was evaluated by comparison with DXA, as the reference method (36, 37). For this purpose, equations were developed (i.e., those described by Mourtzakis et al) (24) to convert the cm^2^ information provided by the CT-scan into Kg provided by DXA. It should be noted that these equations have been validated in subjects with mean BMI<27 kg/m^2^, most of them with cancer and malnutrition. At present there is no data regarding the accuracy of these equations in PwO. Furthermore, these equations were based only on the area parameter (cm^2^) provided by the CT-scan and did not take into the account the HU.

On this basis, we designed the present study aimed to a) evaluate the accuracy of the pre-existing equations (based on cm^2^ provided by CT images), to estimate in Kg the BC in PwO, by comparison with Dual-energy X-ray absorptiometry (DXA). b) evaluate the accuracy of a new approach (based on both cm^2^ and HU parameters provided by CT images), using an automatic software and AI to estimate the BC in kg in PwO, by comparison with DXA.

## Materials and methods

### Patient selection

We performed a single-centre cross-sectional study including consecutive patients with morbid obesity, matched by gender with normal BMI subjects at Vall d´Hebron University Hospital, between April and September 2021. Control group was randomly drawn from patients with DXA performed in our centre with normal BMI (20-25 kg/m^2^).

The study was approved by the local Ethics Committee (PR(AG)510/2021) and carried out in accordance with the Declaration of Helsinki. All the patients signed the informed consent form before the participation in the study.

Inclusion criteria: a) age between 18 and 60 years; b) morbid obesity (BMI >40 kg/m^2^ or BMI > 35 kg/m^2^ with at least one comorbidity related to obesity).

Exclusion criteria: a) any condition except obesity that can affect the body composition, (ex. myopathy, neurodegenerative disease, renal, liver and heart failure etc); b) any treatment that can affect the body composition as per investigator criteria (ex. corticosteroids, growth hormone, etc); c) unable to perform both DEXAand CT scan; d) antropometric data above the usual CT-scan machines (such as body weight >205kg or the presence of an abdominal circumference greater than the ability to obtain an image in a cut (>200cm corresponding to CT-scan gauntry diameter, usually of 70cm); e) metal plates or artifacts that can affect the radiodensity measurements (Hounsfield Units).

### Clinical data collection

All the subjects underwent within a maximum of 30 days from the inclusion in the study: complete medical history, anthropometric data (weight-kg, height-m), biochemical analysis, body composition DXA and skeletal CT centered at L3 vertebrae.

BMI was calculated using the following formula: weight (kg)/height^2^ (m^2^).

### DXA analysis

Body composition DXA was performed using GE Lunar Prodigy dual-energy X-ray absorptiometry (DXA) scanner (GE Healthcare, Madison, WI, USA) by a certified technician. The software used for the total and regional body composition estimation was Encore (GE Healthcare) version 15. Each region was automatically analysed and then supervised by a Nuclear Medicine Physician. The following variables were registered: fat mass (Kg), fat free mass (Kg), appendicular skeletal muscle mass (Kg), appendicular skeletal muscle mass index (Kg/m²).

### CT data extraction

Skeletal CT images focused at L3 vertebrae were obtained using a multidetector computed tomography scanner (Aquilion Prime SP, Canon Medical Systems, Japan), using the following technical parameters: 135 kV (tube voltage), 1mm 80 row (detector configuration), tube current modulation, and 0.8 sec/rotation (gantry rotation). The following variables were registered: skeletal muscle mass area or SMA (cm² and %), skeletal muscle mass index or SMI (cm²/m²), intramuscular adipose tissue area or IMAT (cm² and %), intramuscular adipose tissue index or IIMAT (cm²/m²), area of visceral fat mass (VFA) (cm² and %), subcutaneous fat (SFA)(cm² and %), visceral fat mass index (VFI) (cm²/m²), and subcutaneous fat (SFI)(cm²/m²), and average Hounsfield Units (HU) value per each segmented tissue.

The CT images centered at the third lumbar vertebra (L3) were analysed using FocusedON-BC software. This software presents a user-friendly interface and includes a semiautomatic labeling tool that allows the user to modify the body mass segmentation automatically carried out by the software. To determine skeletal muscle and abdominal adipose tissue area we analysed the cross-sectional CT images at the third lumbar vertebra (L3) using FocusedON-BC software. A total of 16 slices for each patient were assessed. The muscles involved in the analysis were: psoas, erector spinae, quadratus lumborum, transversus abdominis, external and internal obliques, and rectus abdominis muscles. Adipose tissue was classified as subcutaneous, visceral, and infiltrating the muscles. All areas were measured in cm^2^ and normalized for height. Tissue quality was assessed based on its average Hounsfield Units (HU) value. Standard thresholds were employed as follows: -29 to 150 HU for skeletal muscle, -190 to -30 for subcutaneous adipose tissue and -150 to -50 for visceral adipose tissue (32, 38).

### Models used for the body composition evaluation

The research works carried out so far have demonstrated the good correlation between the muscle and fat tissues area, measured in cm^2^ at the L3 vertebral level, with the total amount of muscle and body fat mass, measured in kg by DXA. Mourtzakis et al. (24) has proposed a linear regression model, referred in this work as Model 1, which has also been used by Tewari et al. (39). However, these research works have been carried out using a relatively short number of patients with similar BMI values, and none of them have included people living with morbid obesity. For these reasons, the first goal of this work is to evaluate the suitability of Model 1 to assess muscle mass for patients with a wider range of BMI values, include people living with obesity.

Model 1: This is the most widely used in clinical practice (24) and estimates the fat mass (FM) and fat-free mass (FFM) using the area labelled as fat (visceral and subcutaneous) and muscle in a CT slice at L3 vertebral level (measured in cm²).

This model is described by Equation 1 (Mourtzakis et al. model) (24):


(*eq* 1.*a*) $$FM_{\left(kg\right)}=0.042\cdot \;FM\_CT\_L3_{\left(cm^{2}\right)}+11.2$$

(*eq* 1.*b*) $$FFM_{\left(kg\right)}=0.3\cdot \;Muscle\_CT\_L3_{\left(cm^{2}\right)}+6.06$$

Values of $FM\_CT\_L3_{\left(cm^{2}\right)}$ and $Muscle\_CT\_L3_{\left(cm^{2}\right)}$ for each patient are directly obtained from the FocusedON-BC software. These values were used to estimate the FM and FFM in kilograms using this model.

Model 2: This model has been generated to evaluate if Model 1 methodology is suitable to estimate muscle and fat mass in kg for patients with different BMI values, including people living with obesity. Concretely, least squared method has been used to adjust the linear regression model described in Model 1 (24) to better fit our patient’s data, and hence, better results are expected. This model is described by Equation 2:


(*eq* 2.*a*) $$FM_{\left(kg\right)}=0.058\cdot \;FM\_CT\_L3_{\left(cm^{2}\right)}+7.35\;$$

(*eq* 2.*b*) $$FFM_{\left(kg\right)}=0.27\cdot \;Muscle\_CT\_L3_{\left(cm^{2}\right)}+13.31$$

FocusedON Model: is a new model proposed by our group based on the present study, using FocusedON software. The goal of this approach is to provide a solution that can be used to precisely estimate the body fat mass and fat free mass (in kg) based on the data extracted from a CT scan with independence of the patient BMI. This approach takes in consideration both the tissue area, measured in percentage, and its average density, measured in HU. These data are directly obtained from FocusedON-BC software. This model is described by Equation 3:


(*eq* 3.*a*) $$\rho \approx \frac{HU}{1000}+1\;\;\to \;\;\left(\frac{kg}{cm^{3}}\right)$$

(*eq* 3.*b*) $$FM_{\left(kg\right)}=0.0069\cdot \;\frac{Weight}{\rho_{ROI}}\cdot FAT_{\left(\%\right)}\cdot \rho_{FAT}+4.53$$

(*eq* 3.*c*) FFM (kg)=Weight (kg)−FM (kg)

First, this model considers the tissue density according to its HU average value, as shown in Equation 3.a. This equation estimates the tissue density in kilograms per cubic centimeter based on its radiodensity measured in HU. As FocusedON-BC provides the average patient radiodensity (for the analyzed images), the patient volume can be extrapolated based on this value and the patient weight as $V_{total}=Weight/\rho_{ROI}$ . Then, since FocusedON-BC provides each tissue quantity measured in area percentage, we can extrapolate to estimate the total tissue volume as $V_{tissue}=\;\left(V_{total}\cdot Tissue\;percentage\right)/100$ . Finally, tissue mass measured in kilograms can be estimated according to its density as $M_{tissue}=V_{tissue}\cdot \rho_{tissue}$ . Following this procedure for fat tissue we obtained $FM_{\left(kg\right)}=m\cdot \;\left(Weight/\rho_{ROI}\right)\cdot FAT_{\left(\%\right)}\cdot \rho_{FAT}+n$ . If we replace the weight, density and tissue percentage with our patient data and apply least squared method to calculate *m* and *n* we obtain Equation 3.b. Since we already know patients’ weight and we are estimating the amount of fat mas in kilograms, we can directly know the amount of fat free mass as the difference (Equation 3.c).

### Statistical analyses

Statistical analysis was performed using Python 3.8. Continuous variables are presented as mean ± standard deviation (SD) for normal distributed variables and median ± interquartile range (IQR) for non-normal distributed variables. Categorical variables are presented using percentages. Statistical significance was accepted at p<0.05. Quanti Quanti Plot (QQ) has been used to assess the normal distribution of the dataset. The correlation between analysed methods and the DXA was assessed using linear correlation coefficient (Pearson). Bland-Altman plots were used to estimate agreement between analysed methods and DXA. Additionally, simplified error grid plots have been also used to assess the error obtained in the estimations. The error (<10%, 10-20% or >25%) was measured as the difference between the estimated value and the reference value provided by DXA.

**Sample size calculation**: At present there is no data regarding the use of equations based on CT-scan images for the BC assessment in PwO therefore we were not able to calculate a sample size based on this population. Nevertheless, we took into the account the previous data reported by Mourtzakis (24) for estimating the whole-body fat mass of the subject, measured in kg, based on the fat area quantified in a CT scan image at L3 in cm² was also useful for subjects with a wider range of BMI values, including patients living with obesity. This study obtained the next correlation model using 51 subjects (oncological patients reported by the study with an BMI = 26.9 ± 6.2 kg/m²):


$$\begin{array}{c} FMwhole\;body\;(kg)\;=\;0.042\;\cdot \;FML3\;(cm2)+\\ 11.2\cdot r=0.88,p<0.001 \end{array}$$

Due to the high correlation obtained, and the fact that it has been further validated in other studies (Tewari et al. (39)), we assume that 51 patients should be enough to carry out this proof of concept. However, as we pretended to test the model for subjects with a wider range of BMI values (higher variability), we increased the sample size by 35%, resulting a sample size of 51 x1.35 = 68.85 patients.

Furthermore, we carried out the next calculation (based on Cohen equation) to verify that the sample-size is correct for our goal:


N=Z2·S2d2

N is the sample size; · Z is the critical value of the standard normal distribution at the 5% significance level (1.96); · S² is the estimated standard deviation of the estimating variable (284 cm² of fat tissue measured at L3); · d is the desired precision in the outcome variable (whole-body fat mass measured in kg). Based on this equation, if we expect a precision of 4 kg (d=4), the obtained N value would be also 68 subjects. We included in our study 70 subjects.

## Results

**Figure 1:**
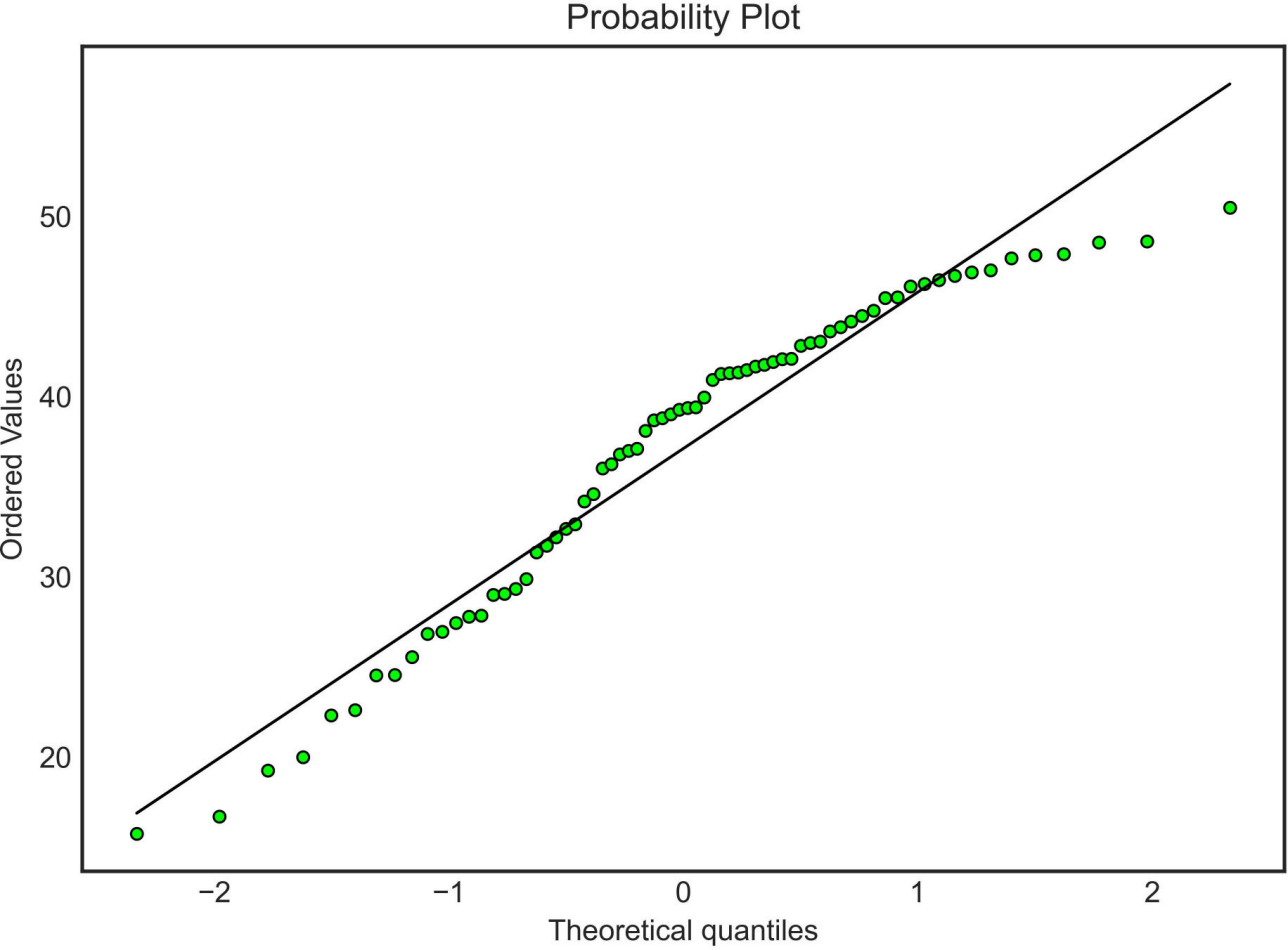
Body mass índex Quanti Quanti Plot.

A total of 70 subjects were included in this study: 46 PwO and 24 with normal BMI. The baseline characteristics are shown in **Table 1**. The BMI of all the subjects, both PwO and normal BMI showed a normal distribution as reflected in the QQ plot displayed in **Figure 1**.

**Table 1:** Demographic and clinical characteristics of the subjects included in the study.

N	All	Patients with MO	Control group	p- value
	70	46	24	
Gender, females, %(n)	59 (41)	70 (32)	38 (9)	0.02
Age (years), mean (SD)	47.37 ± 12.8	43.17 ± 10.35	55.42 ± 13.37	<0.001
Weight (kg), mean (SD)	101.41 ± 23.78	114.37 ± 14.49	76.57 ± 17.5	0.00007
BMI (kg/m^2^), mean (SD)	37.63 ± 9.52	43.56 ± 4.69	26.27 ± 4.86	0.00007

BMI, body mass index; SD, standard desviation.

The correlations and agreement between the reference method, DXA, with the three models that were created for the study are displayed in **Table 2**. Multiple conclusions can be drawn from these results.

**Table 2:** Correlation and agreement between the different models with DXA.

		Fat free mass				Fat Mass			
			Error (kg)				Error (kg)		
		Pearson	-1.96 SD	Mean	+1.96 SD	Pearson	-1.96 SD	Mean	+1.96 SD
**1 slice**	Model 1	0.75	-17.08	-2.76	11.56	0.91	-22.31	-6.03	10.25
	Model 2	0.75	-11.1	3.26	17.62	0.91	-18.56	-4.48	9.59
	FocusedON model	–	-6.77	2.42	11.61	0.93	-11.61	-2.42	6.77
**16 slices**	Model 1	0.76	-16.93	-2.76	11.4	0.90	-22.38	-6.07	10.23
	Model 2	0.76	-10.76	3.64	18.04	0.90	-18.46	-4.38	9.69
	FocusedON model	–	-6.9	2.23	11.36	0.93	-11.36	-2.23	6.9

-, indicates that the fat free mass has not been directly estimated by the FocusedON model, and so, the Pearson coefficient has not been calculated. Instead, the fat free mass has been calculated as the difference between patient weight and the fat mass estimated by the FocusedON model.

First, the correlation and agreement obtained with each model when using only 1 CT slice is very close to the one obtained when using 16 slices. This suggests that a single slice may be representative enough for the BC assessment based on CT scan images.

**Figure 2:**
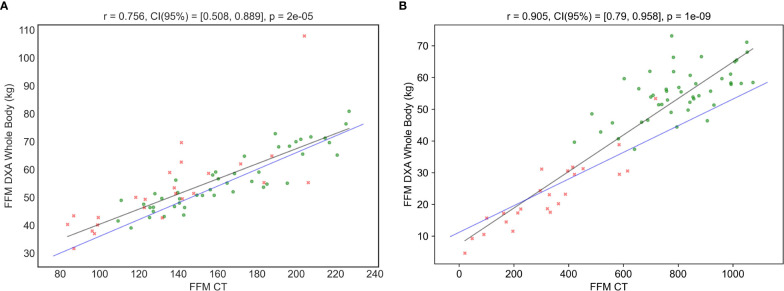
Correlation between DXA and CT in FFM **(A)** and FM **(B)** using the corresponding tissue area in cm2 as TAC Metric. Blue line represents “Model 1”, and black line represents “Model 2”. Red crosses represent the subjects with normal BMI and green dots the subjects with obesity.

**Figure 3:**
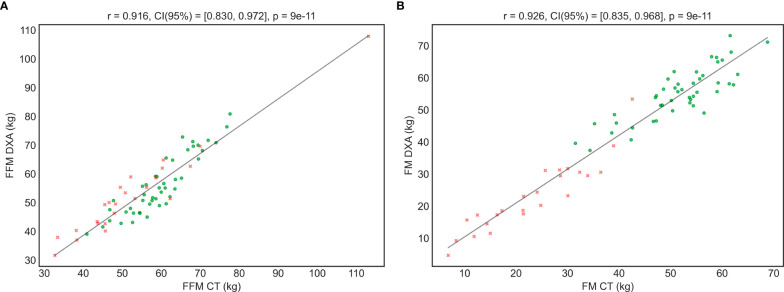
Correlation between the FFM **(A)** and FM **(B)** values measured DXA and using the FocusedON model by TC. Red crosses represent the subjects with normal weight and green dots the subjects with obesity.

Secondly, it can also be observed that all models present high correlation values (higher than 0.75 in all the tests), being **FocusedON Model** the one providing the highest correlation (0.93). It should be noted that a high correlation value does not necessarily imply a good agreement between the reference method, DXA, and the tested model. In this regard, **Figures 2**, **3** graphically show a scatter plot with the trendlines, correlation values and confidence intervals corresponding to each model. In **Figure 2**, where points corresponding to **Model 1** and **Model 2** are considerably far from the tendency, reflects that these two models based on currently used equations, present bad agreement with respect to DXA in PwO. **Figure 3**, that corresponds to **FocusedON Model**, the points are closer to the trendline, showing that the newly proposed model presents a good agreement with respect to DXA.

**Figure 4:**
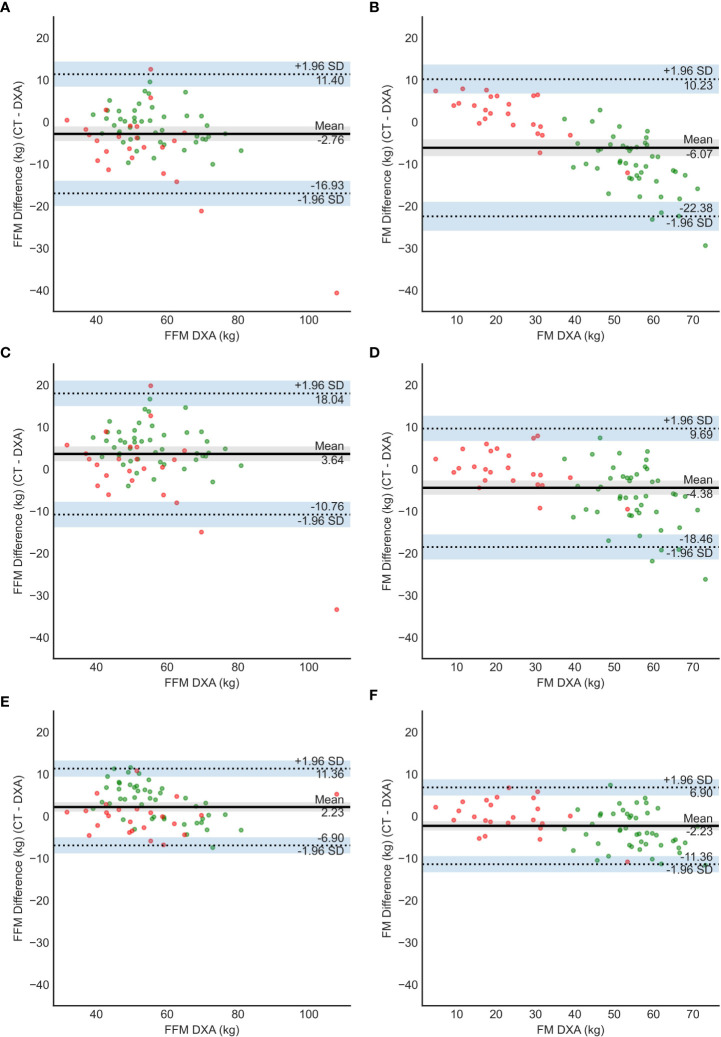
Bland – Altman plots for model 1 **(A, B)**, model 2 **(C, D)** and FocusedON model **(E, F)**. Red dots represent the subjects with normal BMI and green dots the subjects with obesity.

**Figure 5:**
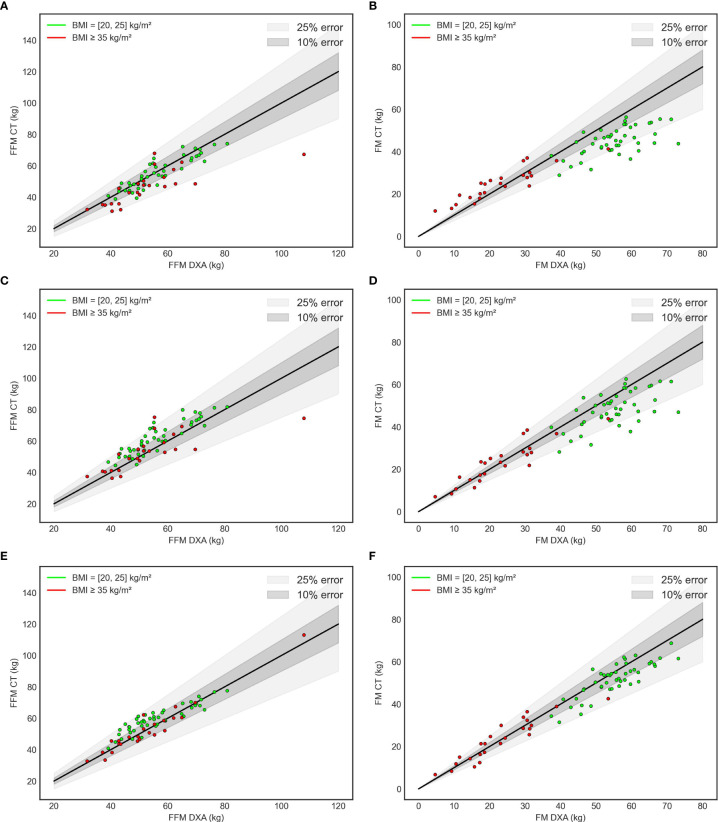
Error grid. Model 1 **(A, B)**, Model 2 **(C, D)** FocusedOn Model **(E, F)**.

Furthermore, for a better accuracy, data was confirmed by two additional statistical analyses (agreement Bland-Adman plots- **Figure 4** and error grid-**Figure 5**). In both analysis, **FocusedON Model** still presented the best agreement with DXA in comparison with Model 1 and Model 2. The numerical values corresponding to these plots are also summarized in **Table 2**.

## Discussion

This is the first study showing that the BC assessment based on skeletal CT images analyzed by automatic software coupled with artificial intelligence, is accurate in PwO, by comparison with DXA. Furthermore, we propose a new equation that estimates both the tissue quantity and quality, that showed higher accuracy compared with those currently used, both in PwO and subjects with normal BMI.

At present, the model most widely used in the clinical practice was developed by Mourtzakis et al. (24). This model estimates the FM and FFM using the area labelled as fat (visceral and subcutaneous) and muscle in a CT slice at L3 vertebral level (measured in cm²). These authors evaluated 51 patients diagnosed of lung or colorectal cancer with a BMI of 26.9 ± 6.2 kg/m^2^ and obtained a high correlation with DXA, r=0.88 (MRE 3.49 ± 2.31 kg, p<0.001) for FM and r=0.94 (MRE 5.23 ± 3.54 kg, p<0.001) for FFM. Few years later Tewari et al. (39) validated the results using the same model, based on 47 patients with esophagogastric cancer with a mean BMI 27.65 ± 5.31 kg/m^2^. They obtained a correlation with DXA of 0.66 (IC 9.451-0.9.964, p<0.0001) for FM and 0.76 (IC 95% -0.8621 – 0.8325, p<0.0001) for FFM. It should be noted that, to the best of our knowledge, most of the studies using this model have been performed in the oncological setting or in critical care units but not in patients with obesity (40–42).

We tested the model proposed by Mourtzakis (24), defined as Model 1, and an adjusted version defined as Model 2 (see Methodology). Bland-Altman plots showed that both models had low agreement with DXA. Both models underestimate the FM for the subjects with obesity and overestimate it in the subjects with normal BMI. In the case of Model 2 the errors were lower but still they remained significant. These findings suggest that these 2 models, based only in the tissue area, measured in cm^2^, cannot be generalized for a wide range of BMIs to accurately estimate body FM and FFM in kg. Additionally, these models are less precise for subjects with obesity.

The CT image also provides the HU of the tissues. The HU is a quantitative measurement of radio density. The absorption/attenuation coefficient of radiation within a tissue is used during CT reconstruction to produce a grayscale image (43). The absorption of X- ray is proportional to the density of the tissue. The use of HU allows to evaluate the tissue quality and estimate its density. In this study we have created a new model that considers, for the first time, both the percentages of adipose and muscle tissue and their average HU, instead of just each tissue area in cm^2^ as in the models used so far in the literature. This new model (Model 3 – FocusedON Model) has provided results that are more accurate and the data was confirmed by three different statistical analysis methods. The correlation between Model 3 and DXA was the strongest. It also showed better agreement with DXA based on Bland-Atman plots, also without under or overestimating the FM and FFM in any of the two groups.

This study proposes a new model, FocusedON model, for estimating body composition based on CT data in PwO and normal BMI. This new model has demonstrated to be superior in accuracy to those presented in previous studies, which only take into the account the quantity of tissue measured in cm^2^. Our model uses parameters of tissue quantity (%) and quality (HU) to create more accurate equations. These findings underlie the importance of revisiting the traditional equations and models where they only used CT measurements expressed in cm^2^.

In addition, it should be noted that the cut-off points commonly used today for the diagnosis of sarcopenia have been developed in oncological population groups, with a small sample size that did not significantly include the overweight or people living with obesity. We have shown in our study that these models are not optimal for the assessment of PwO, so the present cut-off levels should be revaluated. Another novelty resulted from our study is that we propose a simplified method using single-slide CTscan image. Previous study recommends that multiple slices should be analyzed (44). In our study, using multiple slices in the analysis of BC was not significantly superior to use a single slice for the CT-scan image.

Nevertheless, our study has some limitations: First, patient´s weight limit: The method cannot be applied in subjects with severe obesity that cannot undergo CT-scan, such as weight > 200 kg. However, these patients represent<3% of the PwO. Small sample size, it should be noted that we performed a proof-of-concept study with a strong agreement between the model and the reference method. We consider that this apparent limitation has no significant influence on the results of the study due to several arguments: a)the sample size of our study is higher (70 subjects) than the study published by Mourtzakis et al. (24) (51 subjects) as previously explained, which is the currently used model to estimate BC based on CT-scan; b) the proposed model includes not only quantitative but also qualitative variables, allowing a better approximation to the clinical and metabolic reality of the patients. Actually, the combination of these variables will be used in future studies with a larger sample size to produce more complex and precise models (I.e., using non-linear machine learning methods.

In summary, in this proof-of-concept study we demonstrated for the first time that the equations currently used for BC assessment based on CT images are not accurate in PwO. Additionally, we proposed a new simplified model, based on single-slice approach of CT-scan image, including both quantitative and qualitative data of BC, that showed better results than the ones being widely used at present in the clinical practice. Further studies are needed to validate and refine this new methodology, which could open a new avenue in the study and management of obesity, by accurately assess the BC “at a glance” with simple and automatic methods.

## Ethical Approval

All procedures performed in studies involving human participants were in accordance with the ethical standards of the institutional and/or national research committee and with the 1964 Helsinki declaration and its later amendments or comparable ethical standards. This study protocol was reviewed and approved by Comité de Ética de Investigación con Medicamentos del Hospital Universitario Vall d’Hebron, approval number PR(AG)510/2021. The study has been granted an exemption from requiring written informed consent.

## Data availability statement

The original contributions presented in the study are included in the article/supplementary material. Further inquiries can be directed to the corresponding authors.

## Author contributions

Conceptualization: AC, FP, RS. Data curation FP, RG. Formal analysis FP, AC, RG. Funding acquisition RS. Investigation: FP, RG, DE, NR, CE, RB. Methodology FP, RG, AC. Project administration: AC, RS; Resources FP, AC. Software RG. Supervision RS, RB. Validation AC, RS. Visualization AC, RG, DE, CE, NR, RB, RS. Roles/Writing - original draft: FP, RG; Writing - review & editing AC, RS. All authors contributed to the article and approved the submitted version.
